# Differential Gene Expression in Activated Microglia Treated with Adenosine A_2A_ Receptor Antagonists Highlights Olfactory Receptor 56 and T-Cell Activation GTPase-Activating Protein 1 as Potential Biomarkers of the Polarization of Activated Microglia

**DOI:** 10.3390/cells12182213

**Published:** 2023-09-05

**Authors:** Alejandro Lillo, Joan Serrano-Marín, Jaume Lillo, Iu Raïch, Gemma Navarro, Rafael Franco

**Affiliations:** 1 Department of Biochemistry and Physiology, School of Pharmacy and Food Science, Universitat de Barcelona, 08007 Barcelona, Spain; alilloma55@gmail.com (A.L.); lillojaume@gmail.com (J.L.); iuraichipanisello@gmail.com (I.R.); g.navarro@ub.edu (G.N.); 2 CiberNed, Network Center for Neurodegenerative Diseases, National Spanish Health Institute Carlos III, 28029 Madrid, Spain; 3 Molecular Neurobiology Laboratory, Department of Biochemistry and Molecular Biomedicine, Faculty of Biology, Universitat de Barcelona, 08028 Barcelona, Spain; joan.serrano.marin@gmail.com; 4 Institute of Neurosciences, Universitat de Barcelona, 08007 Barcelona, Spain; 5 School of Chemistry, Universitat de Barcelona, 08028 Barcelona, Spain

**Keywords:** adenosine A_3_ receptor, Alzheimer’s disease, microglia, neurodegeneration, neuroinflammation, Parkinson’s disease

## Abstract

Microglial activation often accompanies the plastic changes occurring in the brain of patients with neurodegenerative diseases. A_2A_ and A_3_ adenosine receptors have been proposed as therapeutic targets to combat neurodegeneration. RNAseq was performed using samples isolated from lipopolysaccharide/interferon-γ activated microglia treated with SCH 58261, a selective A_2A_ receptor antagonist, and with both SCH 58261 and 2-Cl-IB-MECA, a selective A_3_ receptor agonist. None of the treatments led to any clear microglial phenotype when gene expression for classical biomarkers of microglial polarization was assessed. However, many of the downregulated genes were directly or indirectly related to immune system-related events. Searching for genes whose expression was both significantly and synergistically affected when treated with the two adenosine receptor ligands, the AC122413.1 and Olfr56 were selected among those that were, respectively, upregulated and downregulated. We therefore propose that the products of these genes, olfactory receptor 56 and T-cell activation GTPase-activating protein 1, deserve attention as potential biomarkers of phenotypes that occur upon microglial activation.

## 1. Introduction

Neurodegenerative disorders cause millions of deaths each year (https://www.who.int/news/item/27-02-2007-neurological-disorders-affect-millions-globally-who-report, accessed on 20 September 2022). In the most frequent neurodegenerative pathologies, Alzheimer’s disease (AD) and Parkinson’s disease (PD), age is the main risk factor. In addition, neuronal death (nigrostriatal dopaminergic neurons in PD, cortical and hippocampal neurons in AD) and protein aggregation are common in these two diseases. Microglia surrounding AD-related pathological structures in human samples show a marked upregulation in microglia of the adenosine A_2A_ receptor (A_2A_R) [1]. Therefore, A_2A_R would be a potential therapeutic target in the event that microglia become a key player in neuroprotection mechanisms.

When they become activated, microglia and macrophages release proinflammatory factors to later undergo phenotypic changes that lead to the expression of molecules that limit inflammation and enable the return to homeostasis. There has been an instrumental nomenclature that assumes a resting M0 state which, upon activation, leads to a proinflammatory M1 phenotype and then results in a resolving M2 phenotype [2,3,4,5]. Another model proposes a continuum of phenotypes as an alternative to the discrete M0/M1/M2 view [6,7]. There is strong evidence of neuroprotective microglia, that is, a phenotype that helps neuronal survival in neurodegenerative scenarios [8,9,10,11]. Current challenges include defining neuroprotective microglia markers and identifying targets to skew microglia toward the neuroprotective phenotype.

Antagonists of the A_2A_R are promising to combat neurodegenerative diseases due to evidence from different laboratories in preclinical research in different animal models of neurodegeneration and because a first-in-class A_2A_R antagonist, istradefylline, has been approved in Japan and the US for human use. Targeting the A_2A_R in microglia is one of the possible mechanisms of neuroprotection. In fact, microglia-mediated neuroinflammation caused by intraperitoneal administration of lipopolysaccharide (LPS) can be prevented by intracerebroventricular administration of SCH 58261, a selective A_2A_R antagonist [12]. Neuroinflammation accompanying the lesion in the 1-methyl-4-phenyl-1,2,3,6-tetrahydropyridine (MPTP) rodent model of PD can also be reverted by intraperitoneal administration of A_2A_R antagonists [13]. Furthermore, A_2A_R antagonists revert microglial activation in a rat model of striatal neurodegeneration [14]. In primary cultures, it is possible to correlate A_2A_R activation with increases in nitric oxide release by activated microglia [15].

Another adenosine receptor, the A_3_ (A_3_R), which has been more recently proposed as a target for neuroprotection, is also expressed in activated microglia [16,17]. There is evidence that the benefits would derive from the activation of A_3_R, that is, neuroprotection would be obtained through the use of A_3_R agonists. Similar effects exerted by A_2A_R antagonists or A_3_R agonists would make sense because the A_2A_R is coupled to heterotrimeric Gs proteins, whose engagement activates adenylate cyclase, whereas the A_3_R is coupled to heterotrimeric Gi proteins whose engagement inhibits adenylate cyclase [18]. Therefore, A_3_R agonists would lower cAMP levels as would A_2A_R antagonists (there is always a certain A_2A_R activation tone due to the continuous presence of extracellular adenosine). The scenario is more complex as the two receptors may establish direct interactions in such a way that A_3_R-mediated signaling is enhanced when A_2A_R antagonists are present [19].

Herein, we aimed to determine by a transcriptomic approach whether (i) simultaneous activation of the A_3_R and blockade of the A_2A_R in activated microglia modulates gene expression, with special attention to genes encoding factors involved in inflammatory responses, and (ii) whether adenosine receptor targeting leads to skewing microglia toward the neuroprotective phenotype.

## 2. Methodology

### 2.1. Reagents

Chloroform (C2432-500ML) was obtained from Sigma Aldrich (St. Louis, MO, USA), Trizol (15696026) was obtained from Ambion Life Technologies (Waltham, MA, USA), isopropanol (131090.1211) was obtained from PanReac AppliChem (Chicago, IL, USA), lipopolysaccharide (L4391-1MG) and human interferon-γ (I3265-1MG) were obtained from Sigma-Aldrich (St. Louis, MO, USA), 2-Cl-IB-MECA (1104), and SCH 58261 (2270) was obtained from Tocris Bioscience (Bristol, UK). After dissolution with dimethyl sulfoxide, adenosine receptor ligands, 10 mM aliquots, were prepared and stored at −20 °C. For use, aliquots were thawed and, unless otherwise indicated, dilution was performed in the culture medium; all conditions, control and treatment with ligands, had the same final concentration of dimethyl sulfoxide.

### 2.2. Isolation and Activation of Microglia

To prepare mice striatal primary microglial cultures, the brains from 2–4-day-old pups (C57/BL6 mice) were used. A total of 4 pregnant animals were used in this study. The procedure was based on previously published protocols [20,21,22]. Tissue was dissected, meninges were removed, and 0.25% trypsin was used for 30 min at 37 °C. Digestion was stopped by adding an equal volume of medium (Dulbecco’s modified Eagle medium-F-12 nutrient mixture, fetal bovine serum 10%, penicillin 100 U/mL, streptomycin 100 μg/mL and amphotericin B 0.5 μg/mL) with 160 μg/mL deoxyribonuclease I. Cells were grown in DMEM medium supplemented with MEM Non-Essential Amino Acids Solution (1/100), 100 U/mL penicillin/streptomycin, 2 mM L-glutamine, and 5% (*v*/*v*) heat-inactivated Fetal Bovine Serum (FBS). Repeated pipetting and passage through a 100 μm pore mesh were followed by centrifugation at 200× *g* for 7 min. The cell pellet was placed in Dulbecco’s modified Eagle medium (DMEM) containing 2 mM L-glutamine (DMEM-g) and seeded in 6-well plates at a 3.5 × 10^5^ cells/mL density; 24 h later, cells were placed in DMEM-g supplemented with MEM Non-Essential Amino Acid Solution (1/100) and 10% (*v*/*v*) heat-inactivated Fetal Bovine Serum (FBS) containing 100 U/mL penicillin/streptomycin. Cultures were maintained at 37 °C in a humidified 5% CO_2_ atmosphere and medium was replaced at DIV 2 and once a week. All cell culture reagents were from Invitrogen, Paisley, Scotland, UK.

Cell viability was calculated by counting alive and dead cells a Countless II FL automated cell counter (Thermo Fisher Scientific-Life Technologies, Waltham, MA, USA) after (1:1 *v*/*v*) dilution with trypan blue. Viability was >98%. The purity of microglial cells upon labeling with anti-CD11b antibodies was >95%.

Cells were cultured for 15 days in a 5% CO_2_ humid atmosphere (37 °C) prior to activation using 0.01% (*v*/*v*) LPS and 0.002% (*v*/*v*) IFN-γ. Vehicle or adenosine receptor ligands were added to the culture at 24, at 32, and, also, at 40 h after the start of the LPS/IFN-γ-induced activation. 48 h after the start of activation, i.e., 8 h after the last addition of vehicle or adenosine ligands, RNA was extracted using Trizol (details in [23]) and purification was performed using isopropanol and chloroform. Purity and integrity were assessed by, respectively, the 280/260 absorption ratio and the RNA Integrity Number (RIN). Further quality control for each sample was performed at the German facilities of Novogene where sequencing was performed. Following mRNA purification using poly-T oligo-attached magnetic beads, first-strand cDNA synthesis was achieved using random hexamer primers and for second-strand cDNA synthesis, dTTP (non-directional library) or dUTP (directional library). For the non-directional library, it was ready after end repair, A-tailing, adapter ligation, size selection, amplification, and purification. The directional library it was ready after end repair, A-tailing, adapter ligation, size selection, USER enzyme digestion, amplification, and purification.

Qubit was used for precise quantification of total RNA, and Bioanalyzer was used for assessing the RNA integrity. Only samples with a RIN > 9 were selected. The number of processed samples (replicates) was 4 for vehicle-treated cells and 3 for receptor ligand-treated cells.

### 2.3. RNAseq Data Processing

Sequences were obtained using Novogene NovaSeq 6000 (pair-end 150), being 151 + 8 + 8 + 151 in the sequencing cycles.

Quality control of sequencing data (raw reads) was first achieved via in-house “perl” scripts. “Clean reads” were obtained upon removal of reads containing adapter or ploy-N; low-quality reads (<Q20) were also removed prior to final analysis. Paired-end clean reads were aligned to the reference genome using Hisat2 v2.0.5.

Feature Counts v1.5.0-p3 software was used for counting the reads to each gene-derived mRNA. Fragments per Kb were calculated considering the length of a given gene and the read count for the gene. Differential expression analysis was done using millions of base pairs sequenced (FPKM) and considering the effect of sequencing depth and gene length for the reads.

For mapping the reads, the reference genome, and gene model annotation files were downloaded from the Ensembl genome browser. The indexing process of the reference genome was carried out using Hisat2 v2.0.5 for indexing the reference genome and for alignment of paired-end clean reads. We selected Hisat2 as the mapping tool because Hisat2 can generate a database of splice junctions based on the gene model annotation file and thus a better mapping result than other non-splice mapping tools.

### 2.4. Differential Expression Analysis

Prior to differential gene expression analysis, for each sequenced library, the read counts were adjusted by edgeR package. Differential expression analysis was performed using the DESeq2R package (1.20.0). The software uses the negative binomial distribution for analysis and determination of *p*-values; the Benjamini–Hochberg approach was employed for assessing the false discovery rate. The “edgeR” R package was used for comparing the expression in two different experimental conditions. Differentially expressed genes were selected by having a false discovery rate (FDR) < 0.05 and a fold change (FC) > |1.5|; FC shown as positive if upregulated and negative if downregulated.

### 2.5. Data Curation

Only coding sequences were considered in the analysis. Sequences coding for proteins that have not been properly characterized were not selected.

### 2.6. Gene Set Enrichment Analyses

STRING is defined as a “*database of known and predicted protein-protein interactions*” https://string-db.org/ accessed on 29 October 2022). STRING v11, which implements well-known classification systems such as Gene Ontology [24], was used for obtaining the potential connections of the differentially expressed genes with the following settings: full network, i.e., considering indirect (functional) and direct (physical) interactions, no additional shells (only the products of the genes provided to STRING were considered) and a confidence of 0.4. Afterward, hits were clustered using the REVIGO online tool (http://revigo.irb.hr/ (accessed on 30 July 2023)), which allows a 2D plot for grouping gene ontologies (GOs) by similarity. The Cytoscape software (v.3.9.1) (https://cytoscape.org/ (accessed on 30 July 2023)) was used to better reveal the results of the comparisons.

To assess the overrepresented transcription factors, the Enrichr tool “interactive and collaborative HTML5 gene list enrichment analysis tool” (https://maayanlab.cloud/Enrichr/ accessed on 29 October 2022) was used, selecting as the curated database the Transcriptional Regulatory Relationships Unraveled by Sentence-based Text-mining (TRRUST) v.2). Afterwards, STRING was also used again for assessing for different interactions between the overrepresented TFs (same settings as those described above).

## 3. Results

### 3.1. RNAseq in Activated Microglia in the Presence of an Antagonist of the A_2A_R

**Figure 1:**
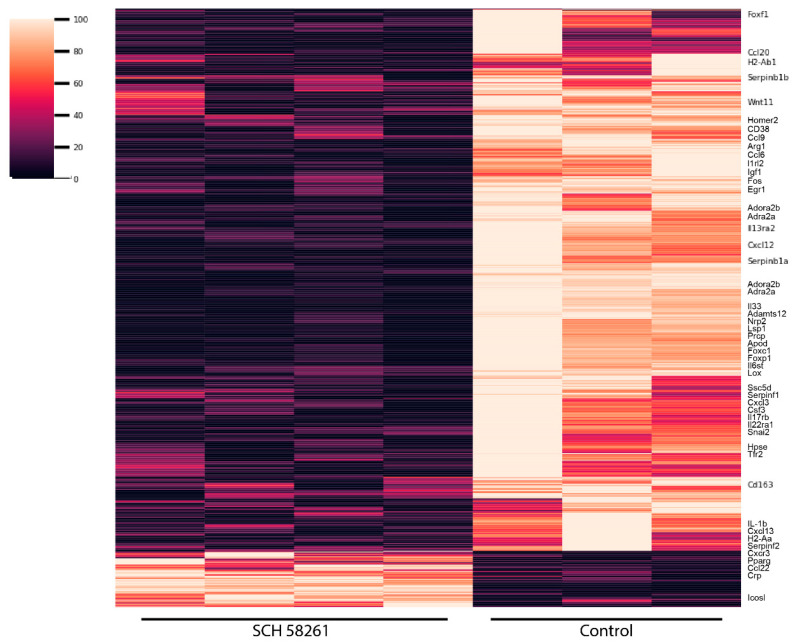
Heatmap of the differentially expressed genes. Only the names of genes related to inflammatory events and cytokine signaling are shown. Heatmaps show only those genes that, when comparing control and antagonist treatment, met the criteria: FC > |1.5| and FDR < 0.05. Lighter colors indicate upregulated gene expression and darker colors indicate downregulated gene expression. Replicates numbered three for vehicle-treated cells and four for receptor ligand-treated cells.

The heatmaps resulting from comparing the expression of genes in activated microglia and activated microglia treated with 200 nM SCH 58261 at 24, 32, and 40 h after the start of the LPS/IFN-γ-induced activation are shown in Figure 1. In all cases, a combination of LPS + IFN-γ was used for activation; cell collection and processing were carried out 48 h after the treatment with 0.01% (*v*/*v*) LPS and 0.002% (*v*/*v*) IFN-γ., i.e., 8 h after the last addition of vehicle or adenosine ligands. Meeting the criteria for a false discovery rate (FDR) < 0.05 and for a fold change (FC) > |1.5|; FC shown as positive if upregulated and negative if downregulated. The number of downregulated genes in cells treated with the A_2A_R antagonist, 1597, was much higher than the number of upregulated genes, 444. The data on the genes whose expression was reliably determined are provided in Supplementary Table S1.

**Figure 2:**
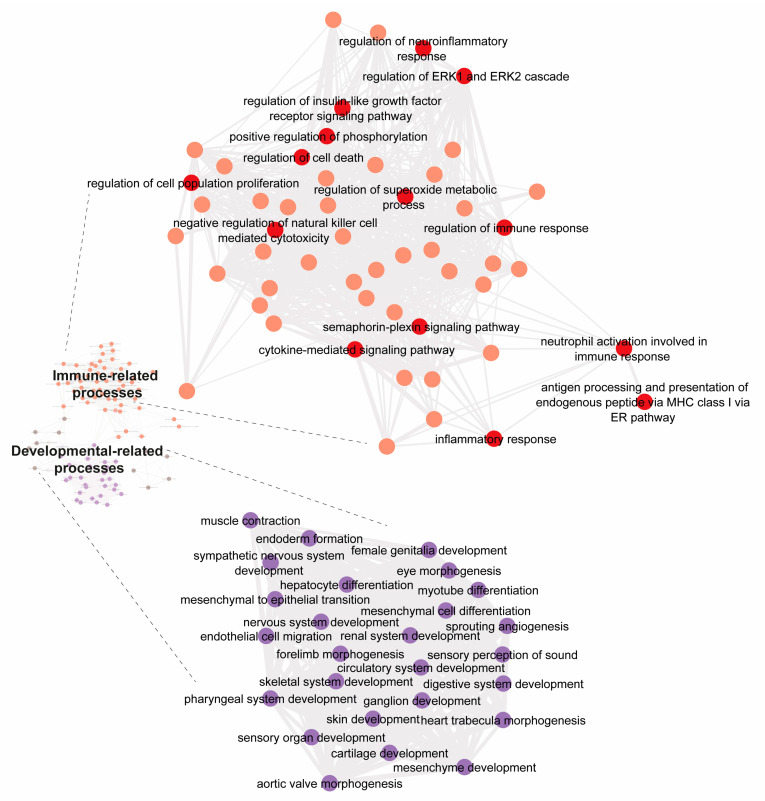
Gene ontology enrichment analysis on the set of transcription factor genes whose expression decreases upon treatment with SCH 58261. Enrichment analysis for genes (for transcription factors) downregulated upon treatment with 200 nM SCH 58261. The two main clusters are highlighted. A more detailed version may be found in Supplementary Figure S1.

Taking into account the genes whose expression was down-regulated upon SCH 58261 treatment, the enrichment analysis leads to data summarized in Figure 2. When analyzing genes whose expression decreases, several GOs (268) were identified (Figure 2); they were mainly clustered within developmental-related and stress/inflammation-related processes. Some of the GOs related to developmental-related processes are mesenchyme development, nervous system development, and endoderm formation. On the other hand, some of the key GOs associated with stress/inflammation are inflammatory response, regulation of immune response, and cytokine-mediated signaling pathway.

**Figure 3:**
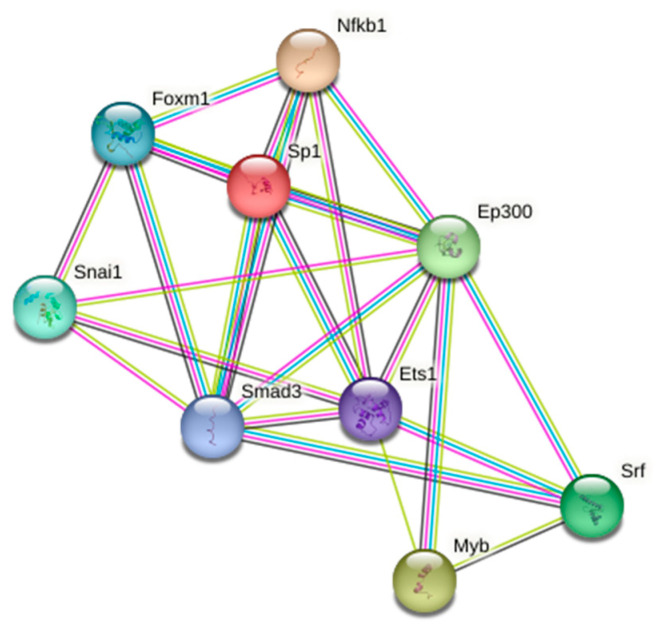
STRING analysis of interactions considering the product of genes whose expression decreases upon A_2A_R antagonist treatment. The colors of the edges represent the following approaches to finding associations: experimentally determined (magenta), gene co-occurrence (blue), co-expression (black) or text mining (lime). Ep300: E1A-binding protein p300; Ets1: ETS proto-oncogene 1; Foxm1: forkhead box protein M1; Myb: myeloblastosis family of transcription factors; Nfkb1: Nuclear factor kappa B Subunit 1; Smad3: SMAD family member 3; Snai1: snail family transcriptional repressor 1; Sp1: specificity protein 1 transcription factor, and Srf: serum response factor.

The genes that were upregulated upon A_2A_R antagonist treatment were not associated with any relevant cell event. In contrast, some of the genes that were downregulated are associated with the functionality of several transcription factors (Table 1) some of which regulate key cellular events (Figure 2). Analysis of known and predicted interactions, taking into account the protein products of downregulated genes, reveals that specificity protein 1 (Sp1) and SMAD family member 3 (SMAD3) transcription factors are nodes connecting two networks. In addition, the nuclear factor kappa B is connected to both Sp1 and SMAD3, whereas Ep300, which is also connected to Sp1 and SMAD3, is related to the myeloblastosis (Myb) family of transcription factors, which are key to inflammatory regulation but have not been characterized in microglia (Figure 3).

**Figure 4:**
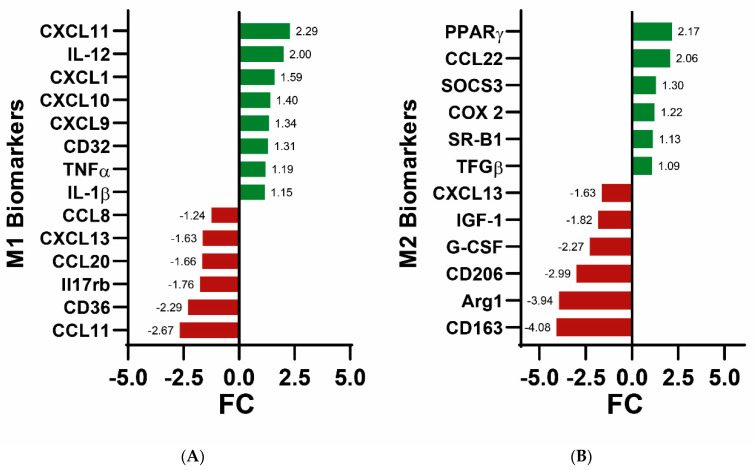
Histogram showing the microglial-phenotype-related biomarkers comparing data obtained in the absence and presence of the A_2A_R antagonist. (**A**) degree of variation (FC) of genes for M1 biomarkers. (**B**) degree of variation (Fold Change; positive if upregulated and negative if downregulated) of genes for M2 biomarkers. Increases in expression due to agonist treatment are in green and decreases are in red. For all these data the FDR was <0.05, that is, genes whose expression was not significantly altered upon A_3_R treatment are not shown. Only genes relevant to M1/M2 polarization are shown.

We addressed the changes in the expression of the genes related to the main biomarkers of M1 and M2 microglial phenotypes (Figure 4). We detected that some biomarkers related to the M1 phenotype were increased (i.e., CXCL11, IL-12, TNFA, IL1-B) and others were decreased (CCL11, CD36, Il17rb). A similar dual pattern was observed regarding the M2 biomarkers. In general, there is no particular trend with respect to microglial polarization, although under the conditions of the A_2A_R blockade here performed, increases in the expression of genes known to be involved in M2 polarization are not obtained, with the exception of peroxisome proliferator-activated receptor γ (PPAR-γ) and CCL22. Therefore, it is not possible to conclude that there is a clear change in phenotype when microglia are exposed to SCH 58261.

### 3.2. RNAseq in Activated Microglia Treated Simultaneously with an Antagonist of the A_2A_R and an Agonist of the A_3_R

**Figure 5:**
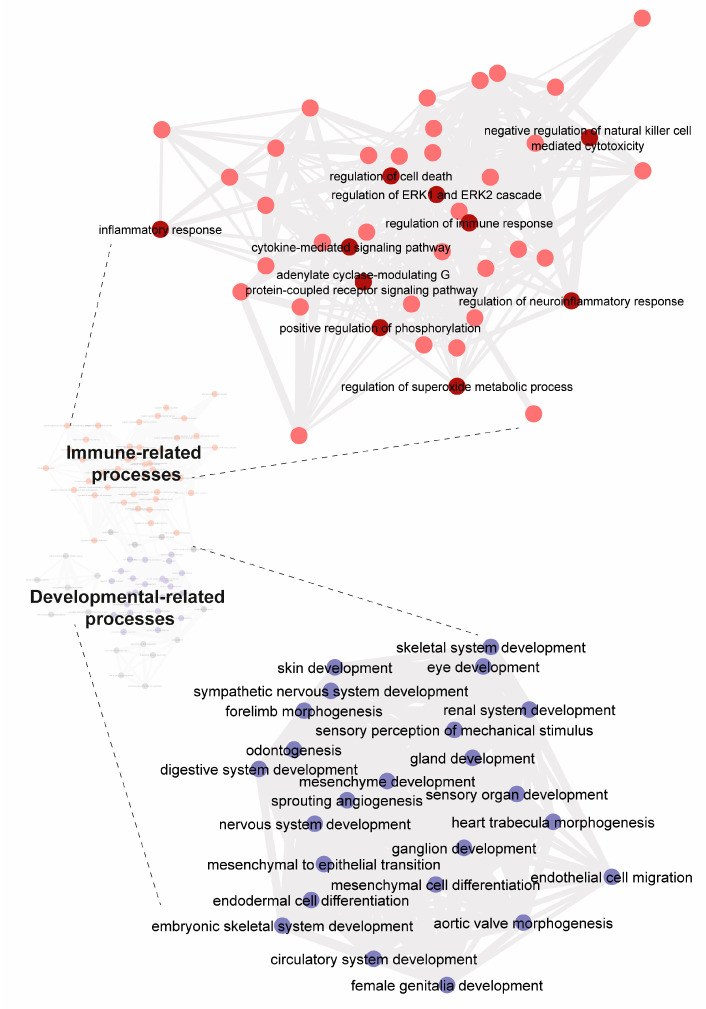
Gene ontology enrichment analysis on the set of transcription factor genes whose expression decreases upon treatment with SCH 58261 and 2-Cl-IB-MECA. Enrichment analysis for genes downregulated upon treatment with 200 nM SCH 58261 and 200 nM 2-Cl-IB-MECA. The two main clusters are highlighted. A more detailed version may be found in Supplementary Figure S2.

Heat maps resulting from comparing gene expression in activated microglia and microglia activated with 200 nM SCH 58261 and 200 nM 2-Cl-IB-MECA at 24, 32, and 40 h after the start of LPS/IFN-γ-induced activation are shown in Supplementary Figure S3. In all cases a combination of LPS + IFN-γ was used for activation; cell collection and processing were done 48 h after the treatment with 0.01% (*v*/*v*) LPS and 0.002% (*v*/*v*) IFN-γ. Meeting the criteria for a false discovery rate (FDR) < 0.05 and for a fold change (FC) > |1.5|, the number of downregulated genes in cells treated with the two adenosine receptor ligands, 1671, was much higher than the number of upregulated genes, 574. Data on the genes whose expression was reliably determined are shown in Supplementary Table S2. All these genes were related to several (222) GOs, which were also mainly related to inflammation-related processes and developmental-related processes (Figure 5).

**Figure 6:**
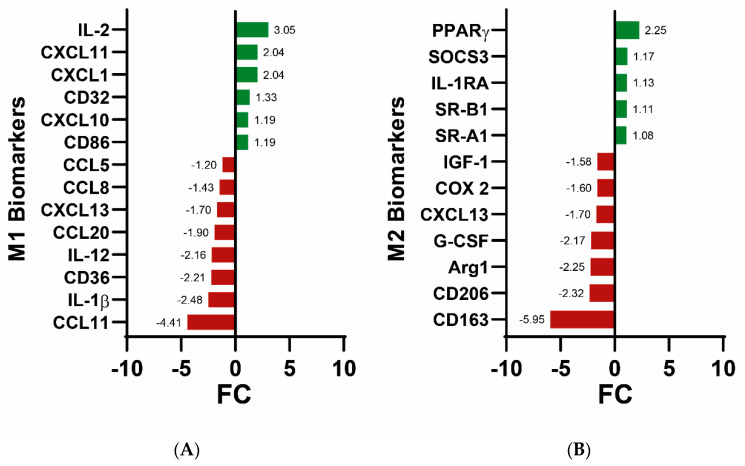
Histogram showing the microglial phenotype-related biomarkers comparing data obtained in the absence and presence of the A_3_R agonist and the A_2A_R antagonist. (**A**) degree of variation (FoldChange-FC-; FC positive if upregulated and negative if downregulated) of genes for M1 biomarkers. (**B**) degree of variation (FoldChange) of genes for M2 biomarkers. Increases in expression due to agonist treatment are in green and decreases are in red. For all these data the FDR was <0.05, that is, genes whose expression was not significantly altered upon A_3_R treatment are not shown. Only genes relevant to M1/M2 polarization are shown.

On addressing the expression of genes coding for biomarkers of M1 and M2 microglia, we found (Figure 6) that, compared with the control samples, the treatment of activated microglia with 200 nM 2-Cl-IB-MECA and 200 nM SCH 58261 led to both upregulation and downregulation of the expression of proinflammatory genes, with the IL-2 being the product of the gene whose expression increased the most, while the chemokine ligand, CCL11, was the product of the gene whose expression decreased in greater magnitude. Regarding M2 biomarkers, PPAR-γ was the product of the gene whose expression increased the most, while the so-called high-affinity scavenger receptor for the hemoglobin-haptoglobin complex, CD163, was the product of the gene whose expression decreased in greater magnitude. Accordingly, there was not any substantial trend regarding M1 and M2 microglial phenotypes.

### 3.3. Comparing RNAseq Data from Individual and Combined Treatments

Aiming at comparing the data obtained in individual versus combined treatment, we decided to select genes whose expression was, at least, 30% different in the combined treatment versus the individual treatment. The curated list of genes whose expression changed more in combined versus individual treatments is shown in Table 2. Genes that were downregulated with the single and combined treatment and for which the downregulation was >30% stronger in the combined treatment were Olfactory Receptor 56 (Olfr56), Amine Oxidase Copper-Containing 3 (Aoc3), ATPase H^+^ Transporting V0 Subunit A4 (Atp6v0a4), ITPR Interacting Domain-Containing 1 (Ccdc129), C-Type Lectin Domain Family 1 Member A (Clec1a), Gastrin-releasing peptide (Grp), indolethylamine N-methyltransferase (Inmt), Keratocan (Kera), and MGAT4 Family Member C (Mgat4c). Only the expression of AC122413.1, a gene that has been related to and associated with selective CA1 neuronal damage in the hippocampus of rodents [25], increased more than 30% in the combined treatment.

To have a more complete set of data for comparison, we retrieved data that were obtained in activated microglial cells treated with 2-Cl-IB-MECA, an A_3_R agonist. RNAseq data from untreated and 2-Cl-IB-MECA-treated activated microglial cells, obtained using the same protocol as the one used here, are deposited in the Gene Expression Omnibus Database (accession number: GSE214330). We listed those genes whose expression was downregulated or upregulated in both the treatment with SCH 58621 and the treatment with 2-Cl-IB-MECA. We then selected those genes and the treatment whose expression in the individual combined treatment was more altered. Finally, we checked the expression of these genes with that of the combined treatment. No threshold was set in FC; the only condition was that the adjusted *p*-value be <0.05. The results appear in Table 2. The genes in the combined treatment that were differentially upregulated taking into account both individual treatments were AC122413.1, Abhd1, Trim17, and Myrip. The genes that in the combined treatment that were differentially downregulated taking into account both individual treatments were Olfr56, Clec1a, Grp, Inmt, Ccdc129, Kera, Aoc3, Ccl11, Small Cajal Body-Specific RNA 2 (Scarna2), Nik-Related Kinase (Nrk), Troponin I1 (Tnni1), Palmdelphin (Palmd), Apolipoprotein F (Apof), and Collagen Type VI Alpha 6 Chain (Col6a6). There was a high decrease in gene expression of an olfactory receptor, Olf56; the decrease was 12.4-fold, 17.9-fold, and 34.5-fold in cells treated with the A_2A_R antagonist, A_3_R agonist, and both compounds, respectively (Table 2). Interestingly, these downregulated genes are uniquely related to the “amine metabolic process” GO (adjusted *p*-value: 0.0014).

## 4. Discussion

The transcriptomic data presented here confirm the expression of functional A_2A_Rs in activated microglia. The results show a large number of genes whose expression is altered by treatment with the selective A_2A_R antagonist; the number of protein-coding genes is reported to be 25,059 in the mouse genome [26]; therefore, the number of genes whose expression is significantly regulated by SCH 58621 (1597 + 444 = 2041) is approximately 8% of the total. The marked effect from A_2A_R blockade demonstrates the relevant role of adenosine in the regulation of microglial activation.

A relevant finding was that increases in gene expression occurred much less than decreases in gene expression. This is similar to what has been recently reported for activated microglia treated with an agonist of the A_3_R [23]. These results seem contradictory from a physiological point of view; in fact, adenosine would apparently act in opposite directions through A_2A_ and A_3_ receptors. The contradiction is not such in cells, such as microglia, that express heteromers of the A_2A_-A_3_ receptor, because A_3_R-mediated signaling is blunted unless an A_2A_R antagonist is present, i.e., those A_3_Rs that are interacting with A_2A_Rs are unable to signal. Actually, this study was undertaken with the hypothesis that A_2A_R antagonists would enhance in activated microglia the action of A_3_R agonists.

The tendency to downregulate rather than upregulate gene transcription suggests that the resulting phenotype reduces the burden associated with maintaining a high degree of gene expression. Transcription factors whose gene expression is regulated by SCH 58621 in activated microglia (Figure 2, Table 1) are involved in almost any cellular event. Gene ontology enrichment analysis shows that regulation of gene expression occurs for several processes, those that are more transversally occurring across cell types and those that are more specific for microglia. The analysis confirmed that several differentially expressed genes participate directly or indirectly in inflammatory processes (Figure 2). The complex set of connections in the immunological/inflammatory GO picture was also found in the co-treatment with the A_3_R agonist and the A_2A_R antagonist (Figure 5).

Despite genes for two M2 biomarkers, PPAR-γ and SOCS3, being upregulated in both treatment with A_2A_R antagonist and combined treatment, the results presented here do not provide any indication that pharmacological manipulation of A_2A_ or A_3_ receptors may lead to M2 polarization, at least using the biomarkers that have been proposed to date. However, strong data on the neuroprotective effect of A_2A_R blockade or A_3_R activation in the scientific literature suggest that there must be M2 biomarkers that have yet to be discovered. Similarly, there is no specific trend regarding M1 biomarkers either using individual or combined treatments. The actual phenotypes may not be well defined based on M1/M2 markers; this would be consistent with the idea that there are microglial intermediate phenotypes [4,6]. As earlier mentioned, it is likely that milder activation protocols would better reflect the reality of in vivo neuroinflammation occurring in patients with neurodegenerative disease, stroke, etc. Our data provide evidence that A_2A_R antagonists and A_3_R agonists can lead to the neuroprotective phenotype, although commonly used biomarkers indicate otherwise. It is likely that it will be necessary to search for new markers of the microglial neuroprotective phenotype. Some of them may be among those featured in the present transcriptomic study.

The gene coding for SMAD3, a transcription factor, was one of the few that were found upregulated upon A_2A_R antagonist treatment. Also notable was the upregulation of NFKB1, the gene encoding another transcription factor, Nfkb1. Both transcription factors regulate transforming growth factor-β (TGF-β) expression and/or TGF-β signaling in diverse types of cells [27,28,29,30,31]. SMAD3 and Nfkb1 appear as multi-connected nodes when the results are rendered with the STRING tool. Despite the fact that SMAD3 has not been, to the best of our knowledge, fully characterized in activated microglia, recent results show that overexpressing the protein (upon transfection) decreases the level of IL-6 and tumor necrosis factor-alpha in microglial BV2 cells [32]. It would be relevant to confirm whether SMAD3 is a biomarker of microglial polarization and define its proinflammatory or neuroprotective role in primary cells activated in physiological-like conditions. Nfkb1 is less attractive, as it is involved in several processes in several cell types (see [33,34,35] for recent reviews).

Other nodes in the STRING enrichment analysis were Sp1, Ets1, Ep300, and Srf; all are related to inflammation linked to neurodegeneration. They could be important to better define the therapeutic effect of A_2A_R antagonists in PD and the potential in the therapy of other neurodegenerative diseases. Sp1 is upregulated in AD mice models [36], and its downregulation in microglia treated with the A_2A_R antagonist could elicit neuroprotective actions. Interestingly, gene polymorphism in the Sp1 gene has been related to the increased risk of developing AD [37]. Ets1 has been also proposed as a therapeutic target to combat diseases associated with neuroinflammatory events [38,39]. Being involved in ROS production [40], the downregulation of this transcription factor could explain, at least in part, the therapeutic effects observed by A_2A_R antagonists in PD. On the other hand, it has been described that Ets1 is targeted by ubiquitination and that its reduction can suppress neuroinflammation [41]

Regarding the Ep300 transcription factor, its downregulation has been proposed as part of the neuropathic pain in rat models [42]. Early microarray correlation studies in the hippocampus of AD patients reported EP300 as an upregulated incipient AD-related gene [43]. The gene codes for Ep300, a cofactor of Creb, whose relevance in both AD and neuroinflammation is known. The Srf transcription factor, which has been linked to inflammation and neurodegeneration in models of epilepsy, is upregulated after an inflammatory stimulus [44,45]. Taking these results into account, the downregulation reported here for gene expression of these transcription factors is consistent with an anti-inflammatory effect of A_2A_R antagonists.

Comparison of the data of single versus double treatment led to interesting findings that are summarized in Table 2. Assuming the hypothesis that both A_2A_R antagonists and A_3_R agonists are neuroprotective and the hypothesis that the combined treatment may have a stronger neuroprotective effect, Table 2 highlights AC122413.1 and Olfr56 as the genes that are more upregulated and downregulated, respectively. Consequently, olfactory receptor 56 could be negatively correlated with the microglial neuroprotective phenotype; in this sense, it would be an inverse marker of neuroprotective microglia. Although the receptor has not been studied in microglia, the interpretation of our result would be in agreement with genome-wide profiling that identifies Olfr56 as a gene that is upregulated (>5-fold induction) in splenic myeloid cells treated with LPS [46]. The AC122413.1 gene becomes very attractive due to the magnitude of its upregulation, >34-fold induction in the combination treatment, and because it encodes a protein that has not been characterized in microglia but in T-cells, T-cell lymphomas, and other cell-related tumor immunity [47,48]. AC122413.1 encodes the T-cell activation GTPase-activating protein 1, which is orthologous to human T-cell activation Rho-GTPase-activating protein (TAGAP). In our opinion, the interest in the possibility that T-cell activation GTPase-activating protein 1 is a marker of neuroprotective microglia is reinforced by the fact that Rho-GTPases are key in numerous cell polarization events. In conclusion, we suggest that the combined evaluation of the expression of Olfr56 and AC122413.1 under conditions of neuroinflammation and neuroprotection would be key to confirming whether or not the protein products of these two genes may be biomarkers for microglial polarization. The severe downregulation of the gene for CCL11, when cells were activated with the two adenosine receptor ligands, should be highlighted. On the one hand, the CCL11 chemokine has been proposed to prevent neurodegeneration [49,50]. On the other hand, excitotoxic neuronal death may be enhanced by CCL11 increasing oxidative stress [51].

A limitation of the study is derived from the assay conditions. One specific issue is the commonly used 48 h activation with LPS and IFN-γ. This robust activation may not reflect actual pathophysiological conditions. It is evident that the presence of serum in cell growth media affects transcription, but the methods of in vitro activation of primary microglia, include serum in the culture medium [52]; therefore, the serum was maintained in all the experimental conditions (control and treatments). Another limitation comes from the fact that the effect of GPCRs is usually studied over short times assuming acute responses. This procedure is very useful, but it ignores the long-term effects that could occur and that depend on the regulation of gene expression. Chronic exposure to drugs that could be useful in neurodegenerative (chronic) diseases is surely affecting gene expression in neurons and microglial cells. A major challenge is how to optimize treatments in primary cultures in terms of concentration of ligands, which can be degraded, and in terms of treatment time because several hours are needed to allow activation of transcription factors and the transcription of up/down-regulated genes. 

In summary, we had to decide the concentrations and timing in the context of microglial activation that is usually triggered by LPS and IFN-γ and lasts for 48 h. Finally, we had to decide the conditions for the treatments with two receptor ligands plus the combination of the two. To mimic the effect of the continuous presence of endogenous adenosine under physiological conditions, we added compounds to cells up to 3 times and waited 8 h between each dose to allow time for gene regulation events to occur. To fully assess the potential to skew activated microglia towards the neuroprotective phenotype, it would be needed to try different doses and find the right time to act, neither too soon nor too late. We consider that it does not make sense to repeat the RNAseq studies in a host of different conditions, that is, we think that despite the limitations, our study is useful to question the validity of the current markers of microglial polarization and encourage research on how olfactory receptor 56 and T-cell activation GTPase 1-activating protein could be involved in the pathophysiology of diseases affecting the CNS. In our opinion, one of the needs arises from another limitation of RNAseq studies, that is, if changes in mRNA expression correlate and translate into changes at the level of protein expression.

## Figures and Tables

**Table 1:** Genes coding for transcription factors whose expression is decreased in cells treated with the A_2A_R antagonist but that are overrepresented after data analysis.

Transcription Factor	Adjusted *p*-Value ^a^	Odds Ratio	Combined Score ^b^
SMAD3	4.67 × 10^−3^	4.7	51.69
ETS1	1.32 × 10^−2^	3.65	32.03
SNAI1	1.92 × 10^−2^	5.27	43.28
SRF	2.15 × 10^−2^	6.83	53.33
SP1	2.15 × 10^−2^	1.93	15.34
EP300	2.87 × 10^−2^	3.25	23.67
NFKB1	4.06 × 10^−2^	2	13.68
MYB	4.86 × 10^−2^	6.38	42.01
FOXM1	4.86 × 10^−2^	5.12	33.32

^a^ Only those with adjusted *p* < 0.05 were considered. ^b^ The combined score is computed by taking the log of the *p*-value from the Fisher exact test and multiplying that by the Z-score of the deviation from the expected rank.

**Table 2:** Genes selected for additive/synergistic effects in the dual treatment.

Gene ID	Gene Name	FC ^a^ A_2A_R Antagonist	FC ^a^ A_3_R Agonist	FC ^a^ Dual Treatment	Ratio ^b^
**Upregulated (FC > 0)**					
ENSMUSG00000116618	AC122413.1	25.51	30.33	34.73	1.15
ENSMUSG00000006638	Abhd1	2.34	2.72	3.63	1.33
ENSMUSG00000036964	Trim17	1.92	1.97	2.31	1.17
ENSMUSG00000041794	Myrip	1.71	1.88	2.13	1.13
**Downregulated (FC < 0)**					
ENSMUSG00000040328	Olfr56	−12.43	−17.88	−34.54	1.93
ENSMUSG00000024517	Grp	−12.84	−9.29	−21.94	1.71
ENSMUSG00000033082	Clec1a	−5.98	−6.70	−12.03	1.79
ENSMUSG00000037973	Ccdc129	−6.40	−3.41	−9.73	1.52
ENSMUSG00000003477	Inmt	−5.90	−8.48	−13.18	1.55
ENSMUSG00000019326	Aoc3	−6.24	−4.41	−8.17	1.31
ENSMUSG00000019932	Kera	−5.75	−3.34	−7.61	1.32
ENSMUSG00000043719	Col6a6	−4.55	−4.78	−6.38	1.33
ENSMUSG00000033377	Palmd	−3.18	−2.63	−4.34	1.37
ENSMUSG00000088185	Scarna2	−2.96	−3.86	−6.88	1.78
ENSMUSG00000026418	Tnni1	−2.94	−3.32	−5.52	1.66
ENSMUSG00000047631	Apof	−2.92	−3.62	−4.83	1.34
ENSMUSG00000052854	Nrk	−2.90	−2.55	−5.10	1.76
ENSMUSG00000020676	Ccl11	−2.67	−3.11	−4.41	1.42

^a^ FC values for the three conditions are indicated: positive if upregulated and negative if downregulated; the reference value for each gene is the expression in microglia that were activated with LPS-IFN-γ but were not treated with adenosine receptor ligands. ^b^ Ratio: value obtained by dividing the FC of the dual treatment by the FC of the individual treatment with the greatest variation; for example, the AC122413.1 ratio, 1.15, results from dividing 34.73 by 30.33.

## Data Availability

Transcriptomics data resulting from this study are loaded into Gene Expression Omnibus database, accession number: GSE222696; data will become public upon paper acceptance. All data used for analysis appear in Supplementary Tables S1 and S2.

## Supplementary Materials

The following supporting information can be downloaded at: https://www.mdpi.com/article/10.3390/cells12182213/s1.
