# Supplementary material for: Differential Gene Expression in Activated Microglia Treated with Adenosine A2A Receptor Antagonists Highlights Olfactory Receptor 56 and T-Cell Activation GTPase-Activating Protein 1 as Potential Biomarkers of the Polarization of Activated Microglia
Source: Cells. 2023 Sep 5;12(18):2213. doi: 10.3390/cells12182213 (PMC10526142; doi:10.3390/cells12182213)
Supplement: Supplementary file 1 [file cells-12-02213-s001.zip › SupplementaryFiguresS1S2_Lillo_Cells.pdf]

Supplementary material.

Supplementary Figures S1 and S2

Differential Gene Expression in Activated Microglia Treated with Adenosine A_{2A} Receptor Antagonists highlights Olfactory Receptor 56 and T-Cell Activation GTPase-Activating Protein 1 as potential Biomarkers of the Polarization of Activated Microglia

Alejandro Lillo, Joan Serrano-Marín, Jaume Lillo, Iu Raïch, Gemma Navarro and Rafael Franco

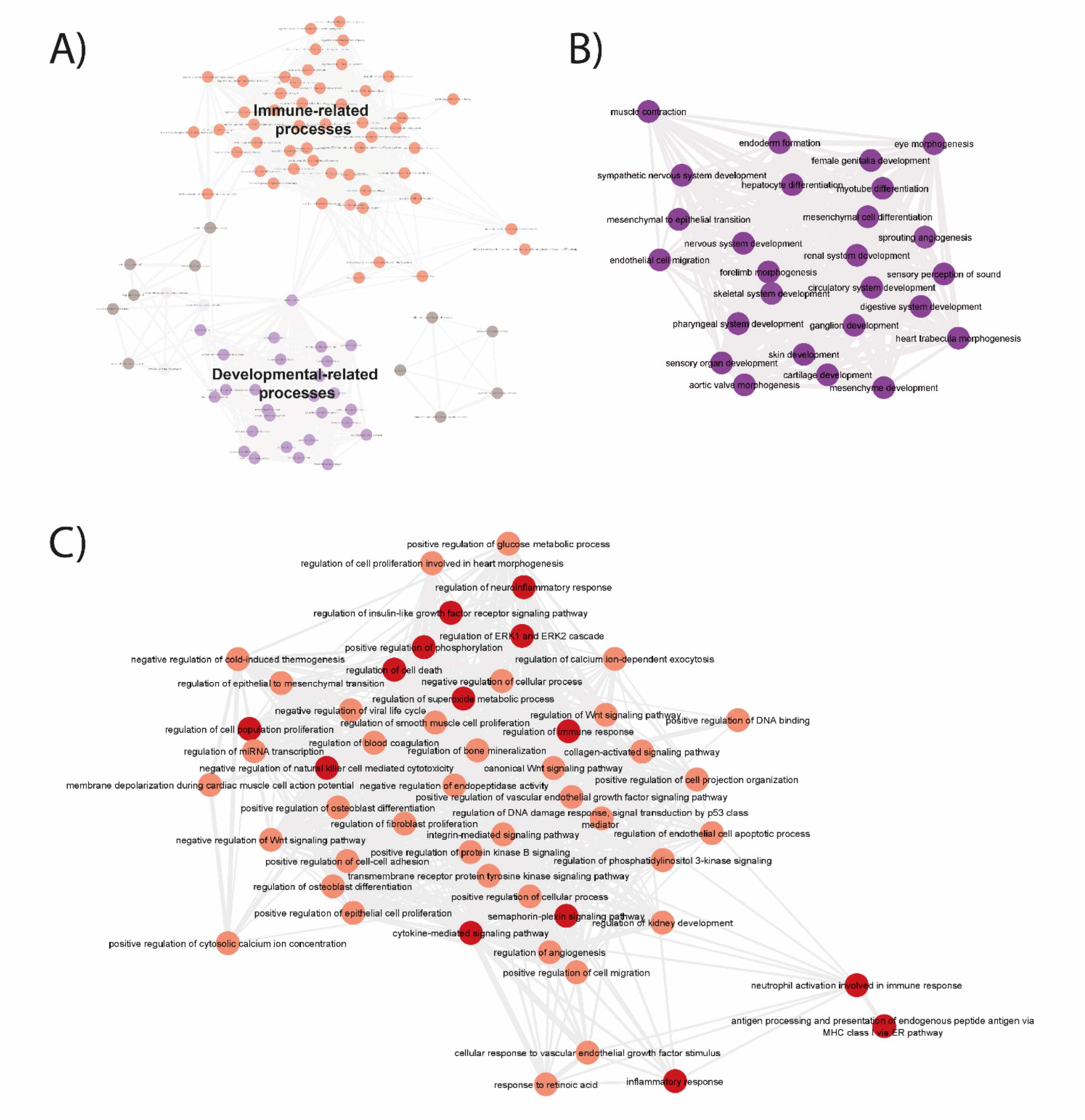

Figure S1. This is the detail of Figure 2 in the paper. Gene ontology enrichment analysis on the set of transcription factor genes whose expression decreases upon treatment with SCH 58261. (**A**) Enrichment analysis for genes (for transcription factors) downregulated upon treatment with 200 nM SCH 58261. (**B**) Developmental-related events in which the genes that downregulated upon $A_{2A}R$ antagonist treatment are involved. (**C**) Immune-related and inflammation-related events in which the genes that downregulated upon $A_{2A}R$ antagonist treatment are involved; dark red indicates direct involvement; light red indicates indirect involvement.

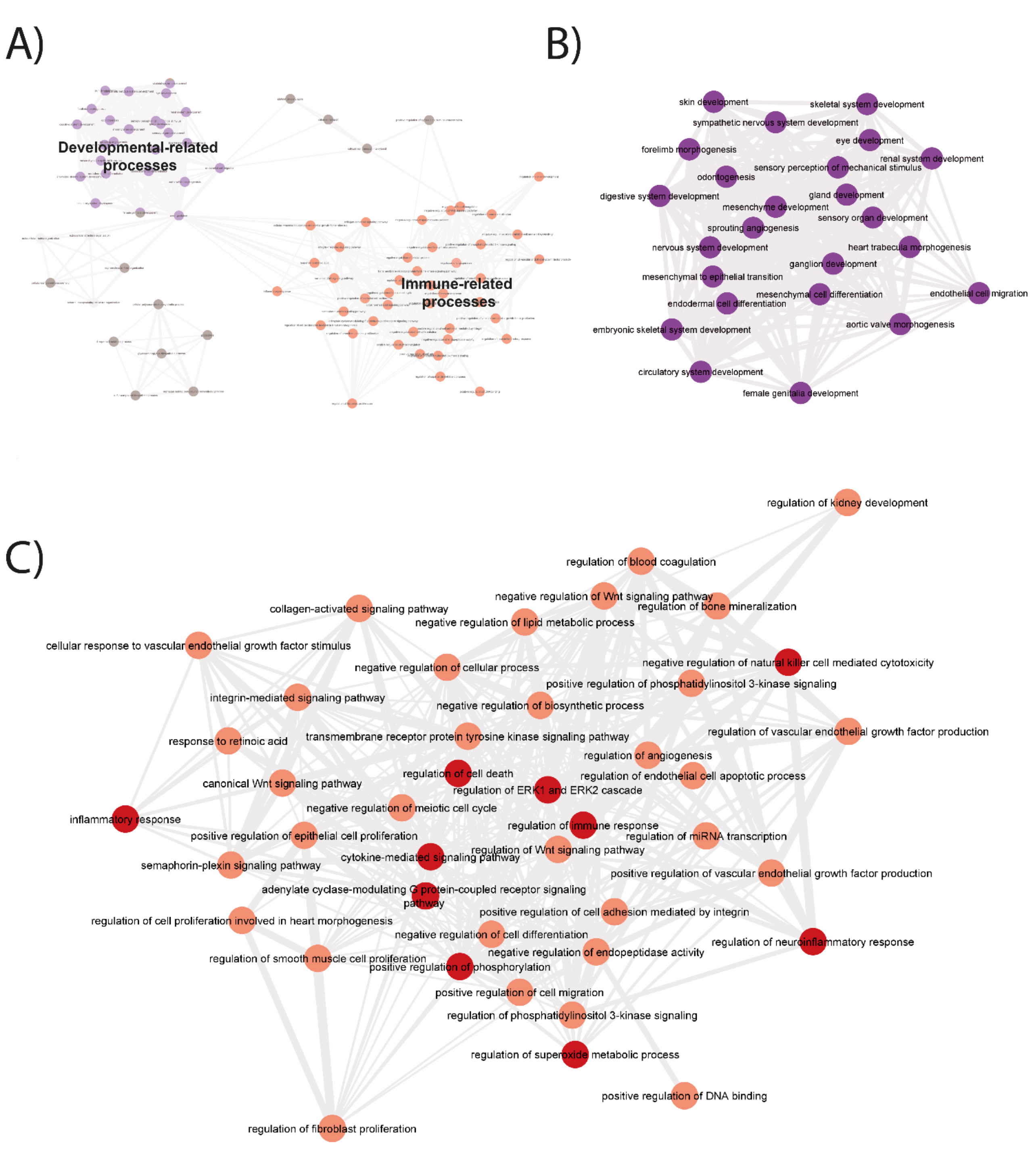

Figure S2. This is the detail of Figure 5 in the paper. Gene ontology enrichment analysis on the set of transcription factor genes whose expression decreases upon treatment with SCH 58261 and 2-Cl-IB-MECA. (**A**) Enrichment analysis for genes downregulated upon treatment with 200 nM SCH 58261 and 200 nM 2-Cl-IB-MECA. (**B**) Developmental-related events in which the genes that downregulated upon treatment with SCH 58261 and 2-Cl-IB-MECA are involved. (**C**) Immune-related and inflammation-related events in which the genes that are downregulated upon treatment with SCH 58261 and 2-Cl-IB-MECA participate; dark red indicates direct involvement in immune system-related processes; light red indicates indirect involvement in immune system-related processes.
