# Supplementary figures and images for: Differential Gene Expression in Activated Microglia Treated with Adenosine A2A Receptor Antagonists Highlights Olfactory Receptor 56 and T-Cell Activation GTPase-Activating Protein 1 as Potential Biomarkers of the Polarization of Activated Microglia
Source: Cells. 2023 Sep 5;12(18):2213. doi: 10.3390/cells12182213 (PMC10526142; doi:10.3390/cells12182213)

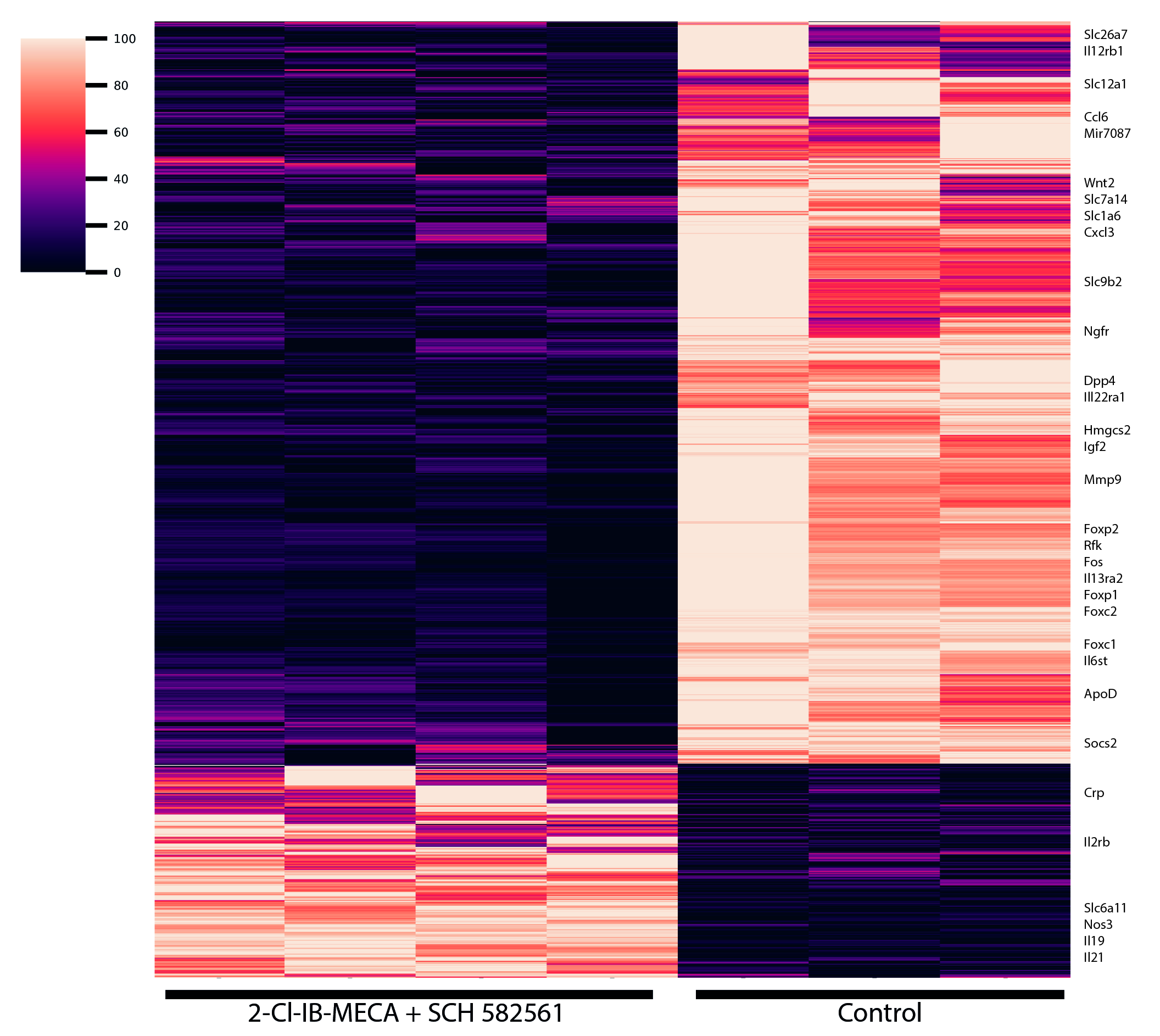

Supplement: Supplementary file 1 [file cells-12-02213-s001.zip › Supplementary_Figure_S3_Lillo_Cells.tif]
